# The MeaB bZIP transcription factor is needed for proper nitrosative stress response induced by nitrite in *Aspergillus fumigatus*

**DOI:** 10.1186/s12864-025-11990-3

**Published:** 2025-09-29

**Authors:** Kinga Edina Varga, Zsigmond Benkő, Károly Antal, Kitti Pázmándi, Zoltán Palczert, István Pócsi, Tamás Emri

**Affiliations:** 1 Department of Molecular Biotechnology and Microbiology, Institute of Biotechnology, Faculty of Science and Technology, University of Debrecen, Debrecen, H-4032 Hungary; 2 Doctoral School of Nutrition and Food Sciences, University of Debrecen, Debrecen, H-4032 Hungary; 3 Department of Zoology, Eszterházy Károly Catholic University, Eger, H-3300 Hungary; 4 Department of Immunology, Faculty of Medicine, University of Debrecen, Debrecen, H-4032 Hungary; 5 HUN-REN–UD Fungal Stress Biology Research Group, Debrecen, H-4032 Hungary

**Keywords:** *Aspergillus fumigatus*, Iron homeostasis, MeaB transcription factor, Nitrosative stress, RNA sequencing

## Abstract

**Background:**

MeaB is a bZIP-type transcription factor in fungi. This protein is known to regulate nitrogen metabolism, contribute to nitrite susceptibility and determine virulence in aspergilli. We hypothesized that MeaB is required for proper nitric oxide (NO) metabolism of fungi. Here, we tested this hypothesis on the human pathogenic fungus *Aspergillus fumigatus* using a transcriptomics approach.

**Results:**

Deletion of the *meaB* gene increased nitrite, diethylamine NONOate and menadione-sodium bisulfite stress sensitivity, but not that of *terc*-butyl hydroperoxide or H_2_O_2_. The conidia of the *ΔmeaB* mutant showed no altered susceptibility to human macrophages. When the transcriptome of the *ΔmeaB* strain was compared with the wild-type strain (Af293), genes involved in siderophore biosynthesis or glucanases were enriched in the up-regulated gene set, whereas genes encoding heme-binding proteins or chitinases were enriched in the down-regulated gene set. The 90 mM NaNO_2_-induced stress elicited a response in the *ΔmeaB* gene deletion mutant that was very similar to that of the wild-type strain in the presence of 135 mM NaNO_2_. These stress responses included the downregulation of mitotic cell cycle and ribosomal protein genes, and the upregulation of nitrosative stress response (*fhpA*,* fhpB*,* gnoA*), nitrate utilization (*niaD*,* niiA*), several iron acquisition and ergosterol biosynthesis genes, as well as the alternative oxidase gene *aoxA*. These stress treatments also altered the transcriptional activity of secondary metabolite cluster and carbohydrate-active enzyme (CAZyme) genes. Nitrite treatment upregulated arginine metabolism genes only in the wild-type strain. The observed transcriptional changes were associated with reduced growth, increased redox imbalance, increased sterol content in both strains and increased nitrite sensitivity of the *ΔmeaB* mutant on arginine as sole C and N source.

**Conclusions:**

Transcriptomic data implies that MeaB affected fine-tuned regulation of arginine metabolism genes, and the alteration in arginine dependent processes (including siderophore production and possibly NO homeostasis) was responsible for the altered phenotype of the *ΔmeaB* mutant. Our results also suggest that, although inhibition of *A. fumigatus* defense against nitrosative stress may not be an effective antifungal therapy for all *A. fumigatus* strains, a combined approach based on disruption of both iron and NO homeostasis is promising.

**Supplementary Information:**

The online version contains supplementary material available at 10.1186/s12864-025-11990-3.

## Background

*Aspergillus fumigatus* is a well-known opportunistic human pathogen [1]. In 2022, the World Health Organization classified this fungus, along with *Candida albicans*, *Candida auris*, and *Cryptococcus neoformans*, as a critical priority group [2]. A better understanding of the biology of *A. fumigatus* may facilitate investment in new antifungal agents, rapid diagnostics, more efficient and accurate identification and susceptibility testing, as well as optimization of existing therapies to improve efficacy, reduce toxicity, and prevent further resistance.

Nitric oxide (NO) is a reactive and readily diffusible compound that acts as an intracellular/intercellular signaling molecule or, when burst, causes nitrosative/nitrooxidative stress in biological systems [3–5]. NO can be synthetized by NO synthases (NOSs) catalyzing the oxidation of arginine to citrulline and NO [6]. NOS-like activity (arginine dependent NO production) has been detected in several fungi including *Aspergillus nidulans* [5, 7], and enzymes with NOS activity were purified from *Saccharomyces cerevisiae* and *Flammulina velutipes* [8, 9]. Nitrite is also an important source of NO in fungi. Nitrite can be reduced directly to NO by mitochondrial nitrite reductases in many fungi [4, 5]; additionally, especially at low pH, nitrite forms NO non-enzymatically [5, 10, 11]. At high concentration, NO can be dangerous. NO can disturb or inactivate metalloproteins by binding to their transition metal [12]. NO can be oxidized by O_2_ to nitrogen dioxide (NO_2_) which can form dinitrogen trioxide (N_2_O_3_) with another NO. NO can react with superoxide (O_2_^−^) producing peroxynitrite (ONOO^−^). These reactive nitrogen intermediates (RNI) can inactivate proteins by forming 3-nitrotyrosine, N-nitrosoamine, and S-nitrosothiol derivatives or oxidizing their thiol-groups and also damage lipids resulting in nitroalkanes [12, 13]. Therefore, NO consuming/detoxifying processes exist in fungi to maintain NO homeostasis and protect themselves from exogenous NO (e.g., when produced by host cells in the case of pathogens) [4, 5]. NO can be reduced to nitrous oxide (N_2_O) by the cytochrome P450-dependent enzyme nitric oxide reductase (Nor) and can be oxidized to nitrate by flavohemoglobin NO dioxygenase (Fhb). NO can also react with glutathione (GSH) forming S-nitrosoglutathione (GSNO), in which case S-nitrosoglutathione reductase (Gno) forms ammonia and oxidized glutathione from GSNO in a NADPH dependent reaction. Alternatively, GSNO S-nitrosylates proteins that are regenerated by a thioredoxin-thioredoxin reductase dependent process [4, 5]. *A. nidulans* and *A. fumigatus* have one Gno (GnoA) and two Fhb (FhpA and FhpB) proteins but Nor has not been identified thus far [14–16].

NO mediated signaling plays multiple roles in fungal life: NO regulates hyphal growth and developmental processes; further, it is involved in the regulation of stress responses, nitrogen metabolism, and secondary metabolite production [4, 5, 17]. In *A. nidulans*, NO is involved in the regulation of conidiation and cleistothecia formation [18–20] and affects sterigmatocystin production [18], while elevated NO production was observed in *A. fumigatus* under heat stress or in the presence of antifungal agents (thymol, farnesol, citral, phenazine-1-carboxylicacid, or pyocyanin) [21]. Due to its multiple and varied physiological/morphological effects, NO also influences virulence and pathogenesis. Besides regulating developmental processes, and mycotoxin production important in pathogenesis, NO produced by fungi can destroy host tissues [4, 5]. NO and nitrosative (nitrooxidative) stress is also dangerous to pathogenic fungi since the attacked host can also generate this stress to protect itself against pathogens [22–24]. However, the importance of nitrosative stress tolerance for virulence is not obvious. Deletion of *fhb1* flavohemoglobin NO dioxygenase gene reduced both nitrosative stress tolerance and virulence in *C. neoformans* [25]. In contrast, the nitrosative stress sensitive *ΔfhpA*, *ΔfhpB*, and *ΔgnoA A. fumigatus* single and double mutants did not show attenuated virulence in mice, and their spores were not killed by macrophages more efficiently than those of the reference strain [15]. While in *C. albicans*, both the null mutant of *YHB1* encoding flavohemoglobin NO dioxygenase, and *CTA4* encoding a transcription factor upregulating *YHB1* under nitrosative stress reduced virulence only moderately in a mouse model, and, in the case of the *YHB1* null mutant, this reduction did not depend on the NOS activity of the host, which suggests that the increased nitrosative stress sensitivity was not responsible for the altered virulence [26, 27]. Similar controversial results were found with oxidative stress tolerance and virulence [28, 29]. These observations may imply that detoxification of RNI (or reactive oxygen species; ROS) are not equally important for the virulence of all pathogenic fungi. Alternatively, the controversial results may be explained by the in vitro determined key elements of stress protection being not necessarily important in vivo, or by the stress developing in vivo in an immunocompromised host being not necessarily restrictive enough to prevent infection [29–31].

A previous study [32] demonstrated that the MeaB bZIP transcription factor contributes to the virulence of *A. fumigatus* (based on the *Galleria mellonella *in vivo infection model). This was explained by MeaB regulating the expression of genes encoding cell wall-related proteins, and thus playing a role in cell wall integrity, tolerance of stresses elicited by cell wall-perturbing agents, and galactosaminogalactan-mediated biofilm formation [32]. Since MeaB has also been described as a regulator of nitrogen metabolism in *A. nidulans* [33–35], here we examined the function of this transcription factor from the view of the utilization/detoxification of nitrite as an important source of NO [4, 5]. We developed a *ΔmeaB* gene deletion mutant from the *A. fumigatus* Af293 wild type strain and used a transcriptomics based approach to better understand its increased NaNO_2_ sensitivity. Ultimately, we suggest that MeaB regulates arginine metabolism in *A. fumigatus.* Thus, the lack of MeaB disturbs arginine metabolism, which has multiple consequences on the physiology of the fungus since arginine metabolism contributes to siderophore production (iron homeostasis), polyamine formation (stress tolerance) and may also affect NO homeostasis. These alteration can contribute to attenuated in vivo virulence in nitrosative stress sensitive strains.

## Methods

### Strains and culturing conditions

*A. fumigatus* Af293 (wild type strain), VKmeaB1 (*meaB* gene deletion mutant of Af293), VKmeaB2 (*meaB* complemented strain of VKmeaB1), and *ΔakuB*^*ku80*^ (parental strain of the *ΔmeaB* mutant used by Chen et al. [32]) [36] were studied. Strains were maintained on Barratt’s (glucose – nitrate) minimal agar plates [37]. Conidia were isolated from 6 days old plates, incubated at 37 °C, with physiological saline – 0.01 v/v % Tween 80 solution and were used immediately to inoculate surface and submerged cultures.

Surface cultures were used to test stress susceptibility of the strains. Barratt’s minimal agar plates were point-inoculated with 5 µL conidium suspension (1 × 10^5^ conidia/mL) in triplicates and were incubated at 37 °C for 5 days. Stress susceptibility was characterized by the reduction in the colony diameter caused by the stressor. The following stressors were studied: CdCl_2_ (2 mmol/L), H_2_O_2_ (0.5 mmol/L), *terc*-butyl-hydroperoxide (tBOOH; 0.8 mmol/L), menadione sodium bisulfite (MSB; 4 and 8 µmol/L), NaNO_2_ (0–60 mmol/L), diethylamine NONOate (DETA NONOate; 2 mmol/L) deferiprone (DFP; 1 mmol/L), Congo Red (CR; 40 mmol/L), sorbitol (1 mol/L), and NaCl (1 mol/L). In some experiments, NaNO_3_ was replaced with 70 mmol/L Na-glutamate, 35 mmol/L arginine, 35 mmol/L ammonium-tartarate, or 4 g/L caseine peptone; glucose or (in the case of DFP treatment) iron was omitted; or the pH was set to pH 4.5 (instead of pH 6.5).

To study the dependence of NaNO_2_ stress tolerance of the mycelium in surface cultures on the nitrogen source, 100 µL conidia suspension (2 × 10^7^ conidia/mL) were spread out on Barratt’s minimal agar plates (pH 4.5) containing either 70 mmol/L NaNO_3_ or 70 mmol/L Na-glutamate as the sole nitrogen source and a 6 mm diameter well was made in the center of each plate. Cultures were pre-incubated for 24 h at 37 °C, then 40 µL of 100 mmol/L freshly prepared NaNO_2_ solution was pipetted into each well and then they were incubated for further 96 h at 37 °C. NaNO_2_ stress susceptibility was characterized by the diameter of the inhibition zone.

Changes in the transcriptome were studied in submerged cultures. Barratt’s minimal liquid media supplemented with 5 g/L yeast extract (100 ml aliquots in 500 ml Erlenmeyer flasks) were inoculated with 5 × 10^7^ conidia. Cultures were incubated at 37 °C and 220 rpm (approximately 3.7 Hz) for 17 h. Mycelia from these exponentially growing cultures were transferred into fresh minimal liquid media containing 11.8 g/L Na-glutamate, 10 g/L glucose, 1.52 g/L KH_2_PO_4_, 0.52 g/L MgSO_4_ 4 H_2_O, and 0.52 g/L KCl and trace elements solution according to Barratt’s et al. [37] (pH 6.5). After a 4 h long incubation at 37 °C and 220 rpm, cultures were treated with 90 mmol/L NaNO_2_, 135 mmol/L NaNO_2_, or were kept untreated, and samples were taken for RNA isolation 0.5 h after treatment.

### Construction of the *meaB* gene deletion and complementation strains

To construct the *meaB* knockout strain (VKmeaB1), the *meaB* (*Afu3g10930*) gene of the *A. fumigatus* Af293 strain was replaced with the hygromycin resistance gene (*hph*) from the pAN7.1 plasmid (AddGene, Watertown, MA, USA). For site-specific recombination, the *hph* gene was associated with approximately 1.5 kb upstream and downstream *meaB* flanking fragments (Table 1).

**Table 1:** Primer pairs used for construction of the *meab* gene deletion and complementation mutants

	Forward (5’−3’)	Reverse (5’−3’)
For gene deletion cassette:		
* php* gene (HiFI_Hyg_F; HiFi_HygB_R)	CGAGCTCCCAAATCTGTCCAGATC	AGCTTGCATGCCTGCAGGTC
upstream region of *meaB *(*meaB*_HiFi_UP_F; *meaB*_HiFi_UP_R)	ATTCTGGTGGAACTGGATGGTGATGTCTAAGCGAGAGGCGTACCAGGG	CATGATCTGGACAGATTTGGGAGCTCGATAGCGACGATGCGCCTGTTG
downstream region of *meaB *(*meaB_*HiFi_DW_F, *meaB*_HiFi_DW_R)	TCCACTCGACCTGCAGGCATGCAAGCTGCGTCGCTGTCCACTTCATTTG	GCCACGCACGGAAAACTTATGACCGTTGAGCATCACGCTTGCCATGTC
gene deletion cassette (*meaB*_UP_F, and *meaB*_DW_R)	CACCTGAGCAGAGTTTTCCAGC	CCTGTCTACAGCCCGCGTAG
For complementation cassette:		
* ble* gene (BleR_F, BleR_001_R)	CTCGTCCACCCCAACGCGTTT	GCCACGCACGGAAAACTTATGACCGTTAACAGTGCAATTATCTTTGCGAACC
* meaB* gene (*meaB*_HiFi_UP_F, *meaB*_Comp_Ble_R)	TCCACTCGACCTGCAGGCATGCAAGCTGCGTCGCTGTCCACTTCATTTG	TGATAAACGCGTTGGGGTGGACGAGAGTGCTCATTGGTGTTGCAC
complementation cassette (*meaB*_HiFi_UP_F, and the BleR_001_R)	TCCACTCGACCTGCAGGCATGCAAGCTGCGTCGCTGTCCACTTCATTTG	GCCACGCACGGAAAACTTATGACCGTTAACAGTGCAATTATCTTTGCGAACC

To complement the deletion of *meaB* in VKmeaB1 (VKmeaB2), a complementation cassette was made by fusing the *meaB* gene, controlled by its own promoter, with the bleomycin resistance gene (*ble*) under the control of the *A. nidulans trpC* promoter, obtained from the pFC333 plasmid (AddGene, Watertown, MA, USA) (Table 1).

For both the deletion and complementation, the appropriate fragments were assembled into the linearized pYTK001 plasmid (AddGene, Watertown, MA, USA) using the NEBuilder HiFi DNA Assembly Kit (New England Biolabs, Ipswich, MA, USA).

Transformation of *A. fumigatus* Af293 was carried out by electroporation of conidiospores with a protocol described previously [38, 39]. A total of 2 × 10^9^ freshly harvested spores were inoculated in 125 ml YG broth containing 5 g/L yeast extract and 20 g/L glucose and were incubated at 37 °C for 4 h at 300 rpm. Swollen spores were collected with centrifugation (5000 g, 10 min, 4 °C), washed once with ice-cold sterile water, re-suspended in 12.5 ml of ice-cold YED (10 g/L yeast extract, 10 g/L glucose in 20 mM HEPES buffer, pH 8.0) and incubated at 30 °C for 1 h, at 100 rpm. Then, spores were collected and re-suspended in 1 ml of 1 mol/L ice-cold sorbitol. 50 µl of the spore suspension was mixed with 1 µg of DNA dissolved in maximum 10 µl H_2_O, incubated on ice for 15 min, then transferred into cuvettes with 0.1 cm electrode gap. The electroporation was performed at 1 kV, 25 µF and 400 Ω. Immediately after electroporation, 1 ml of ice-cold YG broth was added to the mixture, incubated for 15 min on ice, then for 3 h at 37 °C at 100 rpm. The mixture was plated on a minimal media containing 10 g/L glucose, 0.92 g/L ammonium tartrate, 0.52 g/L KCl, 0.52 g/L MgSO_4_ 7 H_2_O, 1.52 g/L KH_2_PO_4_, 1 ml/L Barratt’s trace element solution [37], 20 g/L agar and 250 mg/L hygromycin or 400 mg/L zeocin (pH 6.5), and were incubated at 37 °C for 2 days. The correct integration of the deletion cassette as well as the presence of the complementation cassette were confirmed by PCR. Expression/lack of expression of the *meaB* gene in the VKmeab1 and VKmeaB2 strains was verified by reverse-transcriptional quantitative real-time polymerase chain reaction (RT-qPCR) assay.

### Detecting growth, redox imbalance, and total sterol content in submerged cultures

The increase in the dry cell mass (DCM) was used to characterize growth of submerged cultures. Mycelia from the samples (5 ml) was filtered and dried at room temperature for 2 days before weighing. The redox imbalance induced by NaNO_2_ treatment was detected by either DCF- [40], or DAF-FM assays [21, 41] in cultures incubated either with 10 µmol/L 2’,7’-dichlorofluorescein diacetate for 1 h or with 10 µmol/L 4-amino-5-methylamino-2’,7’-difluorofluorescein diacetate for 0.5 h. Sterol content of lyophilized mycelial samples was extracted with n-heptane after saponification with 25 w/v % KOH dissolved in 65 v/v % ethanol for 1 h at 85 °C. Sterol content was determined spectrophotometrically from the heptane phase [42].

### Susceptibility test of conidia by macrophage killing, generation of human macrophages

Primary monocytes were isolated from human heparinized leukocyte-enriched buffy coat samples using Ficoll-Paque Plus (GE Healthcare, Little Chalfont, Buckinghamshire, UK) gradient centrifugation followed by CD14 microbeads (Miltenyi Biotech, Bergish Gladbach, Germany) based separation. Macrophages were differentiated from primary monocytes as described earlier [40]. Briefly, monocytes were seeded in RPMI 1640 medium (Sigma-Aldrich, St. Louis, MO, USA) supplemented with 10% heat-inactivated fetal bovine serum (FBS, Life Technologies Corporation, Carlsbad, CA, USA), 2 mM L-glutamine (Biosera, Nuaille, France), 100 U/ml penicillin (Biosera, Nuaille, France), 100 µg/ml streptomycin (Biosera, Nuaille, France) for 5 days in the presence of 80 ng/ml granulocyte-macrophage colony-stimulating factor (GM-CSF; Gentaur Molecular Products, London, UK) or 50 ng/ml macrophage colony-stimulating factor (M-CSF; PeproTech, Brussels, Belgium) for M1 and M2 macrophage phenotype, respectively. Differentiated macrophages were seeded in 96-well cell culture plates. To each well, containing 1 × 10^5^ macrophages in 100 µl complex RPMI 1640 medium, 1 × 10^5^ conidia suspended in 100 µl complex RPMI 1640 medium was added. Samples containing 1 × 10^5^ conidia in 200 µl complex RPMI 1640 medium as no macrophage controls were also prepared. After incubation for 4 h at 37 °C in 5% CO_2_ humidified atmosphere, colony forming units were determined on Barratt’s minimal medium as described earlier. Strains (Af293, VKmeaB1, and VKmeaB2) were tested with macrophage phenotypes (M-CSF and GM-CSF) in 8 replicates.

### *Galleria mellonella* infection model

In vivo virulence assays were carried out with *G. mellonella* larvae as described [43]. Briefly, larvae obtained from ZooNet (Budapest, Hungary) were administered with 10 ml of PBS (negative control) or PBS containing 5 × 10^5^ conidia via the last right proleg using an insulin syringe. The survival of the larvae, kept in the dark at 37 °C, was monitored daily for a week. Each group contained 20 randomly selected larvae (weighing 0.2–0.4 g with normal movement and no melanization) and the experiment was carried out in duplicated.

### RT-qPCR assay

Lyophilized mycelial samples were used to isolate total RNA. The protocol of Chomczynski [44] based on Trisol reagent (Sigma-Aldrich, St. Louis, Missouri, USA) was applied. Luna Universal One-Step RT-qPCR Kit (New England Biolabs, Ipswich, MA, USA) were used for RT-qPCR reactions according to the manufacturer’s instructions. See Additional file 1 for the primer pairs applied. ΔCP values were calculated to characterize relative transcription (ΔCP = CP_reference gene_ – CP_target gene_, where CP is crossing point of the reaction). The *tef1* (*Afu1g06390*) gene (putative translation elongation factor EF-1 α-subunit) was selected for reference.

### High throughput RNA sequencing

Illumina sequencing was performed at the Genomic Medicine and Bioinformatic Core Facility, Department of Biochemistry and Molecular Biology, Faculty of Medicine, University of Debrecen, Debrecen, Hungary. Quality of RNA isolated from lyophilized mycelia according to Chomczynski [44] was checked with Eukaryotic Total RNA Nano kit on Agilent Bioanalyzer (Agilent, Santa Clara, CA, USA). For Illumina RNA sequencing (single-read; 75 bp), RNA libraries were prepared with TruSeq RNA Sample preparation kit (Illumina, San Diego, CA, USA) according to the manufacturer’s instructions. All 12 library pools (four types of cultures × three biological replicates) were sequenced in the same lane of a sequencing flow cell. The obtained reads (14–22 million reads/sample) were aligned to the genome of *A. fumigatus* Af293 with hisat2 (version 2.2.1; [45] (Additional file 1). The following reference genome and genome feature files were used: https://fungidb.org/common/downloads/release-65/AfumigatusAf293/fasta/data/FungiDB-65_AfumigatusAf293_Genome.fasta; https://fungidb.org/common/downloads/release-65/AfumigatusAf293/gff/data/FungiDB-65_AfumigatusAf293.gff. The percentage of the successfully aligned reads were between 93% and 94%. Read count values were calculated with FeatureCounts 2.0.6 [46]. Differentially expressed genes (DEGs) were determined with DESeq2 1.40.2 [47].

### Evaluation of transcriptome data

Similarities between transcriptomes were visualized with principal component analysis (PCA) based on rlog values generated by the DESeq2 (version 1.40.2) software.

When two transcriptomes were compared (“A” vs. “B”), DEGs where |log_2_FC| >1 were regarded as upregulated (log_2_FC > 1) or downregulated (log_2_FC < −1) genes. The log_2_FC values were calculated by DESeq2 (version 1.40.2) software with “B” as reference transcriptome.

Upregulated and downregulated gene sets were characterized with gene set enrichment analyses (ShiniGo; bioinformatics.sdstate.edu/go/) using default settings. The enrichment of the following gene groups (Additional file 2) was tested with Fisher’s exact test: “Glycolysis”, and “TCA cycle” (tricarboxylic acid cycle) genes [48]; “Respiration”, “Ribosome protein”, “Ammonia assimilation”, and “Arginine biosynthesis” genes (KEGG pathway database; https://www.genome.jp/pathway/afm00190); “CAZyme” (carbohydrate active enzyme) genes (Carbohydrate-active Enzymes Database, http://www.cazy.org/); “Heme binding protein” gene (FungiFun2; https://elbe.hki-jena.de/fungifun [49],; “Fe-S cluster protein”, “Fe-S cluster assembly”, and “Heme biosynthesis” genes [50], “Iron acquisition” genes [51], “Glutathione-S-transferase” genes (FungiDB, https://fungidb.org); “Antioxidative enzyme”, “Glutathione degradation and synthesis”, “Chitinase”, “Chitine utilization”, “Glucanase” genes [52]; “Squalene-ergosterol pathway” genes [53], as well as several secondary metabolite cluster genes [54, 55].

### Statistical evaluation of the data

Growth values (DCM and colony diameter), redox imbalance (DCF test), sterol content and conidia susceptibility data were presented as mean ± standard deviation (SD) calculated from three (in the case of conidia susceptibility, from 8) biological replicates. Changes in gene expression relative to the reference gene detected by RT-qPCR assays were characterized with ΔCP values and were presented as mean ± SD (*n* = 3). Significant differences between mean values were tested by Student’s *t*-test (two-sided, two-sample test; *p* < 0.05) using Microsoft Excel (2013).

The survival of the *G. mellonella* larvae infected with different strains was tested in duplicated and the data were evaluated with log rank test using the “survdiff“ function of the “survival” package for R project (https://cran.r-project.org/web/packages/survival/index.html).

In the case of high throughput RNA sequencing data, the DESeq2 1.40.2 software [47] was used to identify DEGs and genes with adjusted *p* < 0.05 were regarded as DEG. Three replicates (four and two for Af293 cultures treated with 90 mM and 135 mM NaNO_2_, respectively) were used for these experiments. Gene set enrichment analyses were carried out with DEGs using ShiniGo 0.82 (bioinformatics.sdstate.edu/go/). Hits with a corrected *p* < 0.05 were regarded as significantly enriched. The enrichment of selected gene groups (Additional file 2) in the upregulated or downregulated sets of DEGs was tested with Fisher’s exact test (*p* < 0.05) using the “fisher.test” function of R project (https://www.r-project.org/).

## Results

### The Δ *meaB* gene deletion strain showed increased nitrosative stress sensitivity

The efficient utilization of amino acids is crucial in the pathogenesis of *A. fumigatus*, as demonstrated by the observation that the absence of *mcsA* encoding methylcitrate synthase (involved in amino acid degradation) reduced in vivo virulence [56]. In fact, many nitrogen metabolism genes are known to have direct implication in virulence [57]. NO formation is also related to nitrogen metabolism, and in some fungal species protection against reactive nitrogen species contributes to pathogenicity [25]. MeaB is a transcription factor involved in the regulation of nitrogen metabolism [33–35]. Understanding its function may reveal new details in the pathogenicity of *A. fumigatus*. This is supported by the observation that deletion of the *meaB* gene reduced the virulence of this fungus in vivo [32].

**Fig. 1:**
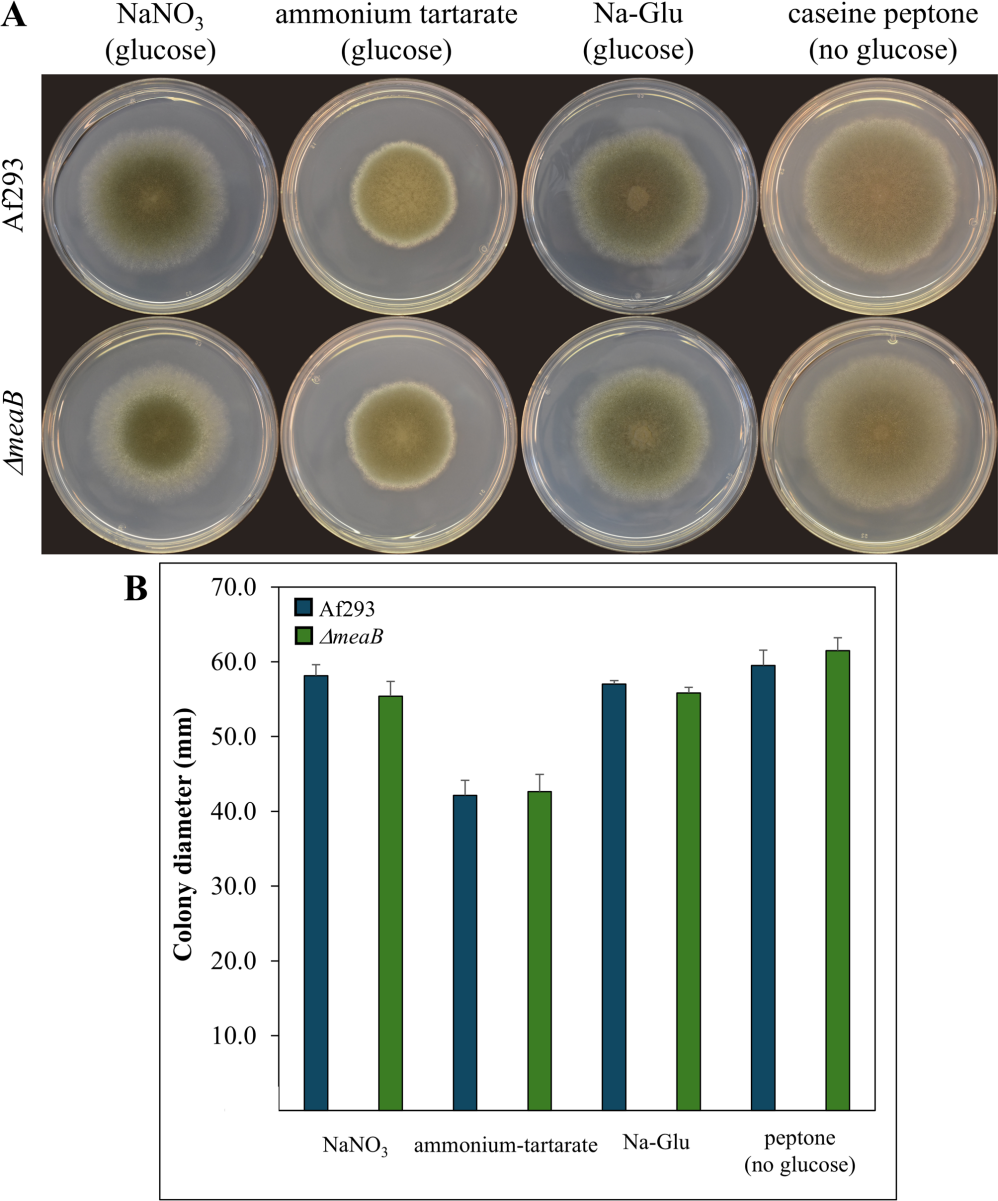
Growth profile of *A. fumigatus* Af293 (wt) and VKmeaB1 (*ΔmeaB*) strains on different nitrogen sources. Panel **A**: Representative photos of 5-day-old cultures. Diameter of the Petri dish used was 85 mm. pH = 6.5. Panel **B**: Colony diameters (mean + SD, *n* = 3). No significant difference (Student’s t-test, *p* < 0.05) was found between the two strains in any culturing conditions

**Fig. 2:**
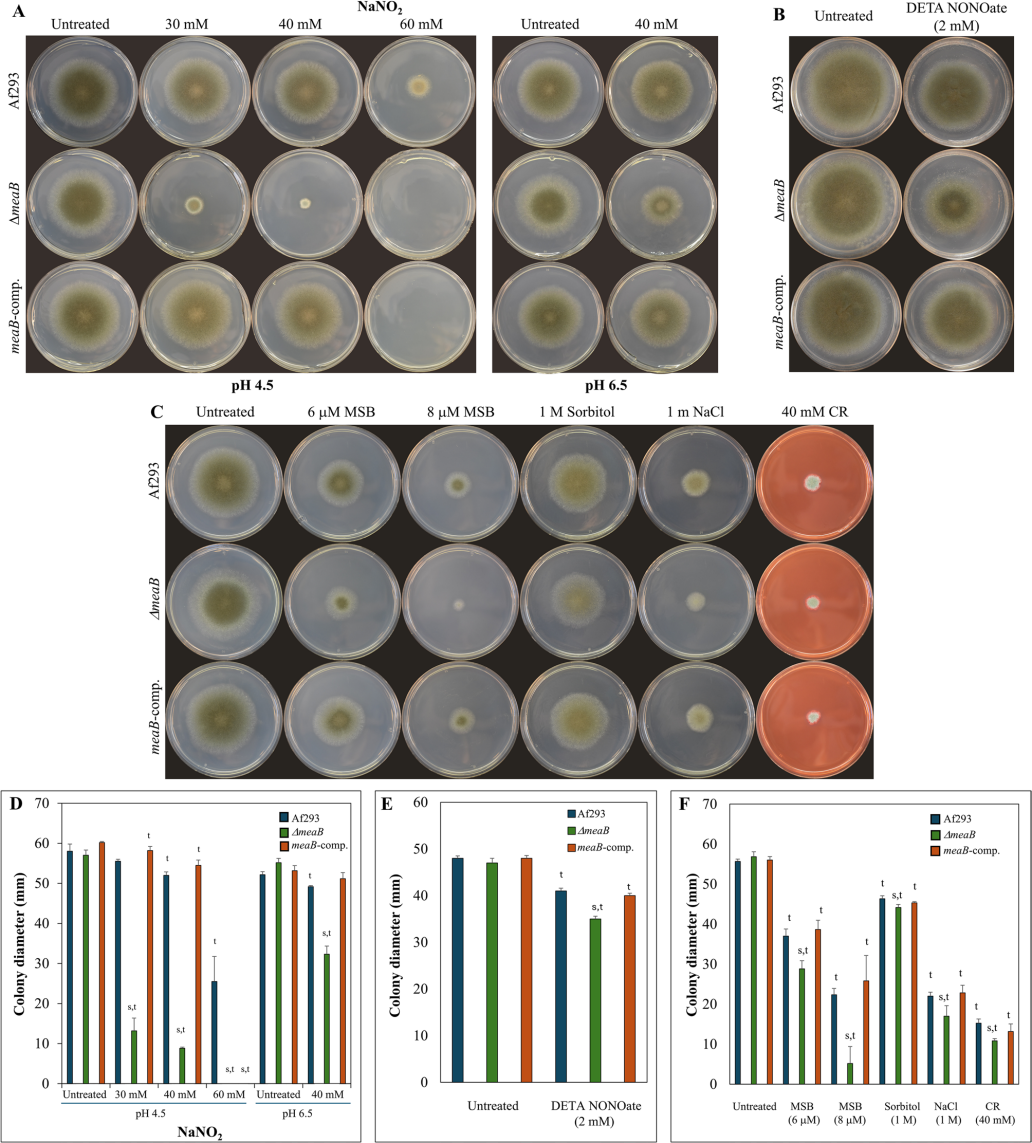
Stress tolerance attributes of *A. fumigatus* Af293 (wt), VKmeaB1 (*ΔmeaB*), and VKmeaB2 (meaB-comp.) strains. Panels **A**-**C**: Representative photos of 5-day-old (Panels **A** and **C**) or 4-day-old (Panel **B**) cultures. Diameter of the Petri dish used was 85 mm (Panels **A** and **C**) or 55 mm (Panel **B**). The carbon and nitrogen sources were glucose and NaNO_3_. (The pH on Panels **B** and **C** was 6.5). Panels **D**-**E**: Colony diameters (mean + SD, *n* = 3). ^t^ – Significant difference (Student’s t-test, *p* < 0.05) was found between stress treated and relevant untreated cultures. ^s^ – Significant difference (Student’s t-test, *p* < 0.05) was found between the mutant (*ΔmeaB* or *meaB*-comp.) and the reference (Af293) strains growing in the same conditions

Deletion of *meaB* had no substantial effect on growth on selected nitrogen sources including nitrate in surface cultures (Fig. 1). Nor did *meaB* gene deletion affect the utilization of casein peptone when it was applied as the sole nitrogen and/or carbon source (Fig. 1). The lack of MeaB increased the sensitivity of *A. fumigatus* against NaNO_2_ (Fig. 2) as was also found earlier with *A. nidulans* [33]. The growth inhibitory effect was pH-dependent, suggesting the importance of RNI formed from NO_2_^−^ especially at acidic pH [10]. Notably, the *Δaku*^*KU80*^ strain, the parental strain of *ΔmeaB* mutant used by Chen et al. [32], was more sensitive to nitrite than the Af293 strain (Additional file 3). Deletion of *meaB* decreased the tolerance against DETA NONOate and MSB as NO and superoxide generating agents (Fig. 2), but had no effect on stress sensitivity elicited by tBOOH, H_2_O_2_ (oxidative stresses), CdCl_2_ (heavy metal stress), or DFP (iron limitation/chelation stress) (Additional file 4). Lack of MeaB decreased CR (cell wall stress), sorbitol, and NaCl (hyperosmotic stress) tolerances as well (Fig. 2), however the changes were not as obvious as found by Chen et al. [32] who used strains derived from *A. fumigatus* A1160 rather than Af293.

Despite of the increased oxidative (MSB) and nitrosative (NaNO_2_, DETA NONOate) stress sensitivity (Fig. 2), the conidia of the *ΔmeaB* mutant did not show altered susceptibility to human M-CSF and GM-CSF macrophages and only showed a small (non-significant) attenuation in virulence using the *G. mellonella* model (Additional file 5). This is in line with the observation that mutants sensitive to nitrosative stress (such as *ΔfhpA*, *ΔfhpB*, or *ΔgnoA* gene deletion mutants originated from the *ΔakuB*^*KU80*^ strain) did not show reduced virulence in mice or increased susceptibility to killing by macrophages [15]. In contrast, Chen et al. [32] found reduced virulence of the *ΔmeaB* gene deletion mutant (also originated from the *ΔakuB*^*KU80*^ strain) in *G. mellonella in vivo* infection model. This difference can be the consequence of the different stress sensitivity of the parental *A. fumigatus* strains (*Δaku*^*KU80*^ and Af293) (Additional file 3) used by Chen et al. [32] and in this study.

### NaNO_2_ treatment disturbed iron homeostasis

**Fig. 3:**
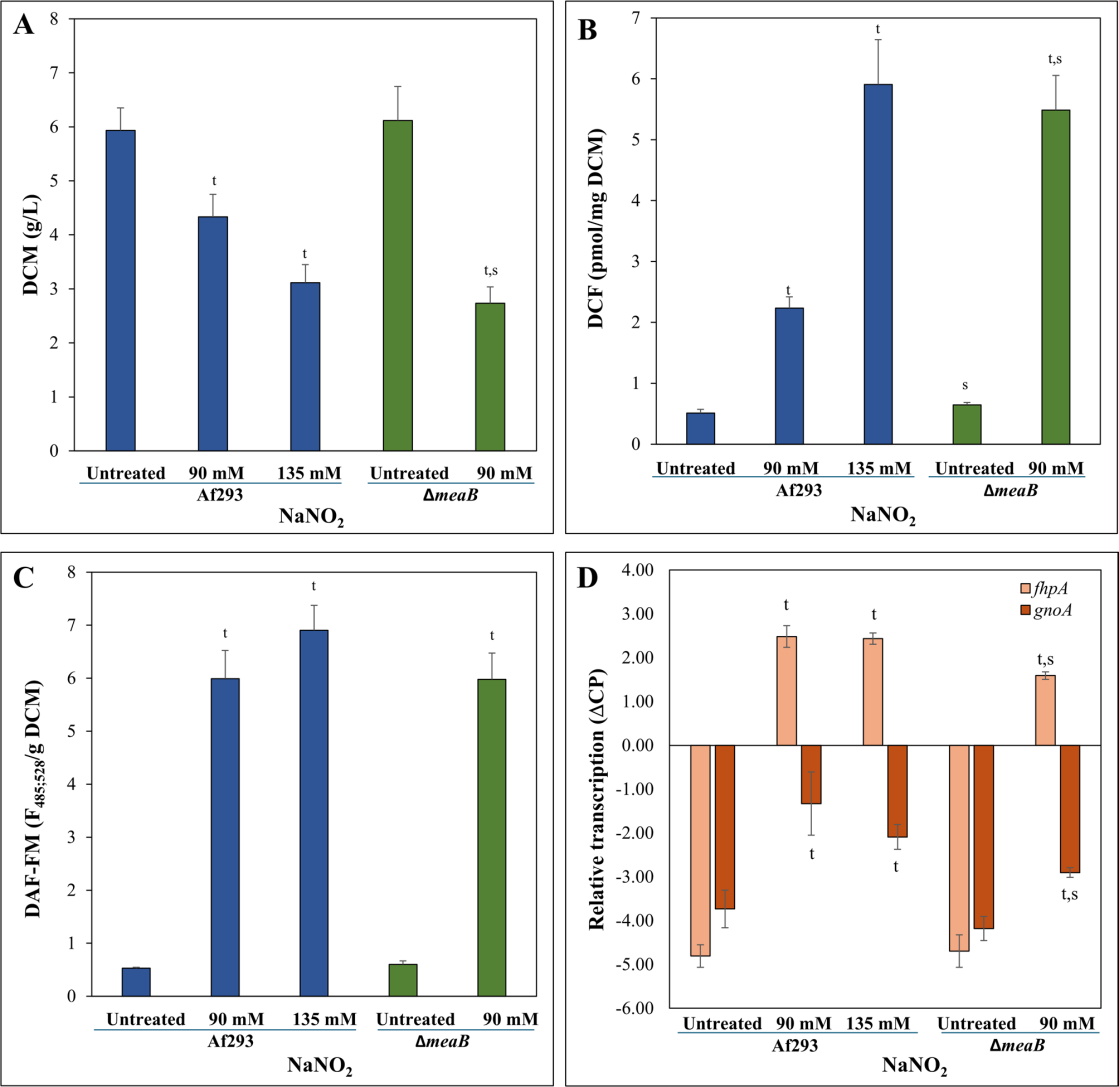
Characteristics of NaNO_2_ treated submerged cultures of *A. fumigatus* Af293 (wt) and VKmeaB1 (*ΔmeaB*) strains. Panel **A**: Changes in the dry cell mass (DCM). Panel **B**: Changes in the redox homeostasis detected by the DCF-test. Panel **C**: Changes in the redox homeostasis detected by the DAF-FM test. Panel **D**: Relative transcription of *fhpA* (light brown) and *gnoA* (dark brown) genes In all panels, mean + or ± SD calculated from three biological replicates are presented. ^t^ - Significant difference (Student’s t-test, *p* < 0.05) between treated and relevant untreated cultures. ^s^ -Significant difference (Student’s t-test; *p* < 0.05) between the two strains under 90 mM NaNO_2_ treatment

For a deeper understanding the link between MeaB and nitrite stress tolerance, we compared the transcriptomes in submerged cultures treated (or not) with NaNO_2_ in the presence of glucose and Na-Glu as carbon and nitrogen sources, respectively, using the *ΔmeaB* gene deletion mutant and the Af293 reference strain. The PCA showed that transcriptomes from the biological replicates were similar within the same (strain and treatment) group, and were significantly affected by treatments for both strains (Additional file 1). We also determined the transcription of 10 genes by RT-qPCR and found a good correlation (Pearson’s correlation coefficients ranged from 0.85 to 0.99) between the transcriptional changes measured by RT-qPCR and RNAseq experiments (Additional file 1). The treatments reduced the growth of the cultures (Fig. 3A), disturbed the redox homeostasis (Fig. 3B and C), and upregulated the key genes of nitrosative stress response such as *gnoA* and *fhpA* [15], encoding S-nitrosoglutathione reductase and cytosolic flavohemoglobin, respectively (Fig. 3D), supporting the view that NaNO_2_ elicited nitrosative stress.

**Fig. 4:**
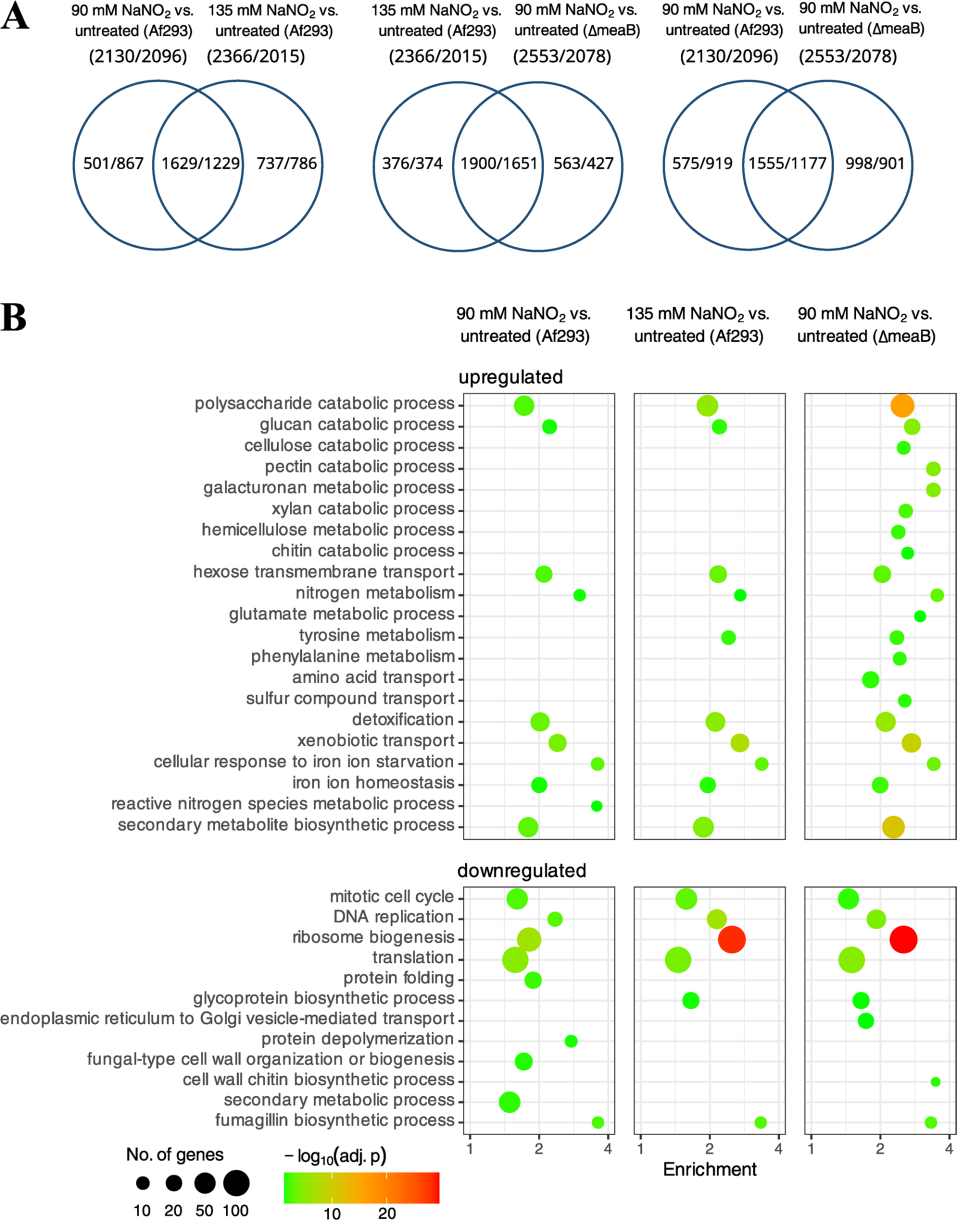
Consequences of NaNO_2_ treatment on the transcriptome of *A. fumigatus* Af293 (wt) and VKmeaB1 (*ΔmeaB*) strains. Part **A**: Venn analyses of the detected upregulated/downregulated genes. Part **B**: Bubble plot representation of selected significantly enriched KEGG pathway and biological process GO terms. Further data on the significantly enriched gene groups are available in Additional files 2 and 6

The 90 mM NaNO_2_ elicited stress response had the following characteristics in the case of the reference strain (*A. fumigatus* Af293):

The detected growth reduction (Fig. 3A) was accompanied by the enrichment of certain vegetative growth-related genes in the downregulated gene set including “Mitotic cell cycle”, “Replication”, “Translation”, “Ribosome biogenesis”, “Ribosome protein”, “fungal-type cell wall organization or biogenesis”, or “Glycolysis” genes (Fig. 4, Additional files 2 and 6).

Despite the detected redox imbalance (Fig. 3B), bulk upregulation of antioxidative enzyme genes was not observed (Additional file 2). Certain, less studied elements of the oxidative stress response, however, showed upregulation: e.g. *fgacat*, but not *catA*, *cat1* and *cat2* [58] catalase genes, *Afu5g02300*, but not the *ccp1* cytochrome c peroxidase gene, and *sod3* but not *sod1*, *sod2*, *sod4* [39] superoxide dismutase genes (Additional file 2). Upregulation of *yap1*, but not *skn7* or *atf1* [59], oxidative stress response regulator transcription factor genes was also observed (Additional file 2).

While the *meaB* gene was upregulated, upregulation of other transcriptional regulators of nitrogen metabolism such as *areA*, *areB*, *tamA*, and *cpcA* [57] was not found (Additional file 2). Even the *nmrA* gene, which is regulated by MeaB in *A. nidulans* [34] did not show upregulation under nitrite stress in *A. fumigatus* (Additional file 2). These data also support the view that NaNO_2_ treatment caused nitrosative stress rather than alterations in nitrogen utilization of the fungus.

**Fig. 5:**
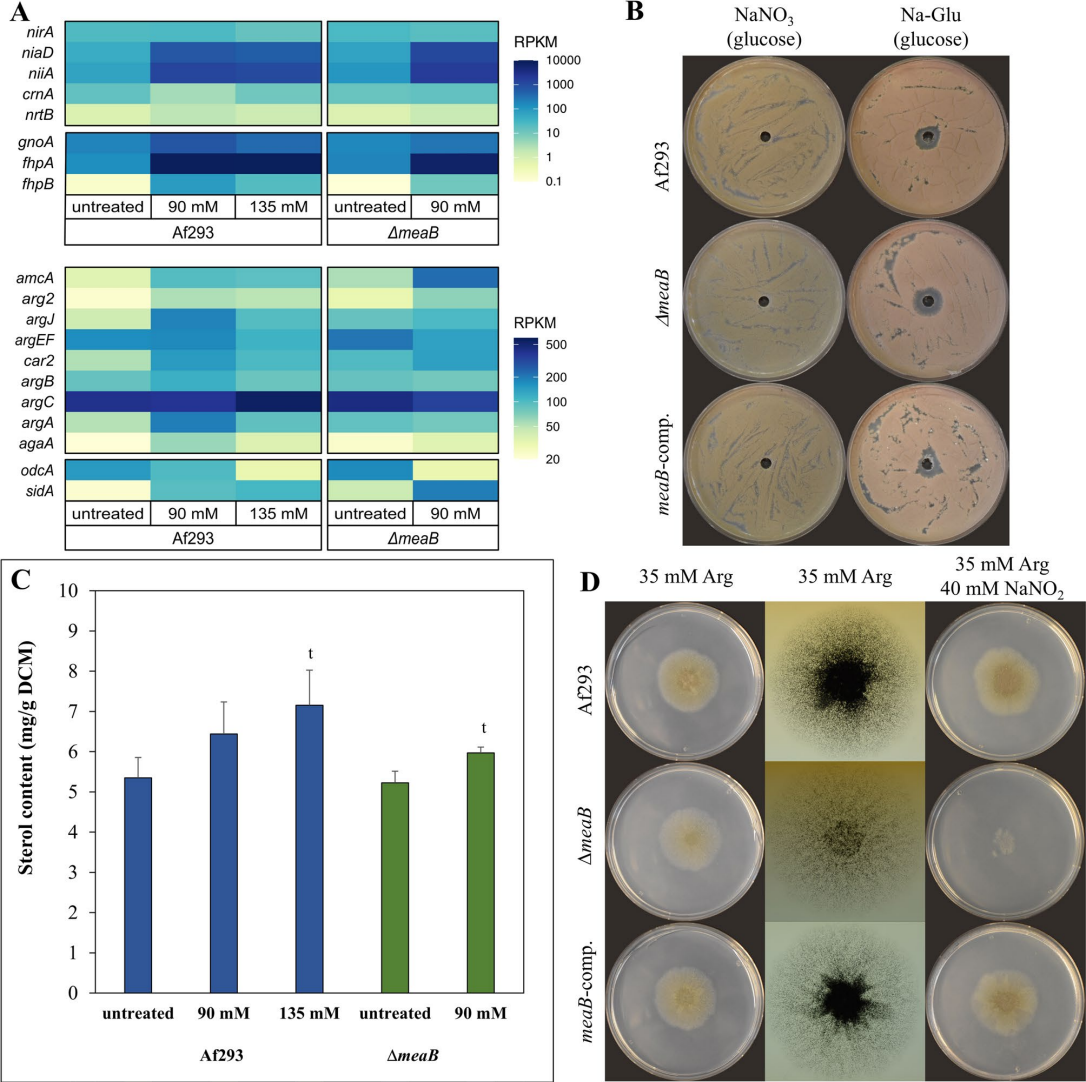
Characteristics of NaNO_2_ treated cultures of *A. fumigatus* Af293 (wt) and VKmeaB1 (*ΔmeaB*) strains. Panel **A**: Heatmap representation of the mean RPKM values of selected genes related to nitrate utilization (*nirA*, *niaD*, *niiA*, *crnA*, *nrtB*), detoxification of RNI (*gnoA*, *fhpA*, *fhpB*), arginine metabolism (*amcA*, *arg2*, *argJ*, *argEF*, *car2*, *argB*, *argC*, *argA*, *agaA*) as well as the key genes of polyamine (*odcA*) and siderophore (*sidA*) biosynthesis. Panel **B**: Nitrite toxicity in *A. fumigatus* cultures growing on nitrate or Na-Glu as sole nitrogen source. Strains were pre-cultured on medium containing either NaNO_3_ or Na-Glu as sole nitrogen source. After 24 h, cultures were treated with NaNO_2_ and the formation of the inhibition zone was recorded at the 5th day. Representative photos are presented. The diameter of the Petri dish was 85 mm. Panel **C**: Changes in sterol content of the strains after NaNO_2_ treatment. Mean + SD calculated from three biological replicates are presented ^t^ - Significant difference (Student’s t-test, *p* < 0.05) between treated and relevant untreated cultures No significant difference (Student’s t-test; *p* < 0.05) was found between the two strains under untreated or NaNO_2_ treated conditions. Panel **D**: Effect of NaNO_2_ treatment in the presence of arginine at pH 6.5. Representative photos of 5-day-old cultures are presented. Diameter of the Petri dish used was 85 mm. Photos in the second column are taken on a transilluminator to visualize the density of conidiophores

Upregulation of *niaD* (nitrate reductase) and *niiA* (nitrite reductase) was also observed (Fig. 5A, Additional file 2). In accordance with the behavior of nitrogen metabolism transcriptional regulators mentioned above, these upregulations were not accompanied with the upregulation of *nirA* (transcriptional activator of nitrate utilization [60], *crnA* (nitrate transporter), or genes of ammonia assimilation (i.e., genes encoding glutamine synthase, glutamate synthase, and NADP dependent glutamate dehydrogenase) (Additional file 2, Fig. 5A). However, “Arginine biosynthesis genes” were enriched in the upregulated gene set (Additional file 2). Upregulation of *niaD* and *niiA* seems to aim detoxification of nitrite to ammonia rather than utilization of it as a nitrogen source. Note, NiiA reduces nitrite to ammonia; NO is oxidized to nitrate by FhpA and nitrate is reduced to nitrite by NiaD [4, 5]. The high transcriptional *niaD*, *niiA* and *fhpA* activities (Fig. 5A) therefore may contribute to complete reduction of nitrite to ammonia, preventing the enzymatic/non-enzymatic formation of NO from nitrite [10] and enzymatic formation of N_2_O from nitrate [4]. In line with this, active nitrate utilization (preincubation with nitrate) reduced the toxicity of nitrite in surface cultures (Fig. 5B).

Nitrite treatment altered iron homeostasis (Additional file 2): “Iron acquisition” genes were enriched in the upregulated gene sets. These upregulated genes include the *fetC*,* frtA* reductive iron assimilation (RIA) genes, elements of the Siderophore (but not the SidC) cluster, as well as *amcA* mitochondrial ornithine carrier protein (supporting siderophore biosynthesis by ornithine [61], and *mirC*, *sit1* siderophore transporter genes (Fig. 5A, Additional file 2). Interestingly, despite the opposite functions of the HapX transcription factor (activates iron uptake genes under iron limitation [51]), and the SreA transcription factor (represses iron uptake genes under sufficient iron supply [51]), both of their genes (*hapX* and *sreA*) were upregulated. Heme binding protein genes (39 genes out of the studied 121 genes) were also enriched in the upregulated gene set (Additional file 2), however substantial transcriptional changes in the case of heme biosynthesis, Fe-S cluster assembly or Fe-S cluster protein genes were not observed (Additional file 2). This is consistent with the observation that NO (generated by NO_2_^−^) can inhibit heme-proteins [62]. Regarding iron-dependent processes, neither ergosterol biosynthesis nor TCA cycle and respiration genes were enriched in the upregulated gene set (Additional file 2). Only the upregulation of *aoxA* encoding alternative oxidase is notable (Additional file 2).

Nitrite treatment altered the transcription of secondary metabolite cluster genes as well. As found previously with other types of stresses [50, 52, 63], the transcriptional activity of some clusters was upregulated, while that of other clusters was downregulated. In the present experiments, genes of the Gliotoxin, Trypacidin, and Afu5g10120 clusters were enriched in the upregulated gene set, while genes of Fumitremorgin B, Fumagillin, and Pseurotin A clusters were in the downregulated gene set (Additional file 2).

Genes encoding CAZymes were enriched in the upregulated gene set (Additional file 2). This upregulation was accompanied with the upregulation of hexose/glucose transporter genes (Fig. 4, Additional file 6). The function of the encoded proteins were diverse and did not link to well-defined carbohydrates (Additional file 2). This pattern resembled those found with carbon starved cultures which secrete CAZymes and other catabolic enzymes to discover alternative carbon sources in their environment (“scouting enzymes” [63]).

According to changes in the DCM and redox homeostasis (Fig. 3A and B), stress elicited with 135 mM NaNO_2_ was stronger than with 90 mM, as expected. The two stress responses were, however, similar: The number of stress responsive upregulated and downregulated genes was not very different, and the overlap between the stress responsive gene sets was substantial (Fig. 4). Accordingly, the stress responsive gene sets were characterized by the enrichment of very similar gene groups (Fig. 4, Additional files 2 and 6). The direct comparison of the transcriptomes of the two NaNO_2_ treated cultures revealed that glucose utilization genes, lipid biosynthesis genes, siderophore cluster genes, Cazyme genes, glutathione S-transferase (GST) genes, and ammonia assimilation genes showed higher transcriptional activity, while ribosome biogenesis genes showed lower transcriptional activity in the 135 mM treated cultures than in the 90 mM treated group (Additional files 2 and 6). Ergosterol biosynthesis genes were also enriched in the upregulated genes set (Additional file 2) and the total sterol content of the cultures increased significantly in the presence of 135 mM NaNO_2_ (Fig. 5C).

### Lack of MeaB also disturbed iron homeostasis even under untreated conditions

NaNO_2_ treatment caused nitrosative stress in the *ΔmeaB* strain too. This treatment reduced growth (Fig. 3A), disturbed redox homeostasis (Fig. 3B and C), upregulated *gnoA* and *fhpA* (Figs. 3D and 4), but bulk upregulation of antioxidative enzyme genes was not observed (Additional file 2). Deletion of *meaB* increased NaNO_2_ sensitivity in submerged cultures (Fig. 3) as it was found earlier in surface cultures (Fig. 2). The PCA of the transcriptome data (Additional file 1) as well as the Venn and gene set enrichment analyses of the up- and downregulated gene sets (Fig. 4, Additional files 2, 6 and 7) demonstrate that in the presence of 90 mM NaNO_2_, the *ΔmeaB* strain behaved very similarly to the 135 mM NaNO_2_ treated Af293 cultures. Mitotic cell cycle, Replication, Ribosome biogenesis and Ribosome protein genes, as well as genes of Fumitremorgin B, Fumagillin, and Pseurotin A clusters were enriched in the downregulated gene set (Fig. 4, Additional files 2 and 6). The *gnoA*, *fhpA* nitrosative stress response genes together with *niaD*, *niiA* nitrate assimilation genes as well as the *yap1* transcription factor gene were upregulated (Fig. 5A, Additional file 2). CAZyme genes and Trypacidin, Gliotoxin, Fumipyrrole, Afu5g10120, Afu3g13730, and Afu3g14700 secondary metabolite cluster genes were enriched in the upregulated gene set (Fig. 4, Additional files 2 and 6). Although ergosterol biosynthesis genes were not enriched in the upregulated gene set (*p* = 0,05858; Additional file 2), NaNO_2_ treatment increased the total sterol content of the hyphae significantly (Fig. 5C). Hexadehydro-astechrome cluster genes, Chitinase, but not Chitin (oligomer) utilization genes, and Glucanase genes were also enriched in the upregulated gene set. These genes did not show enrichment in the case of the Af293 strain. However, direct comparison of the transcriptomes of treated cultures (90 mM NaNO_2_, *ΔmeaB* vs. 135 mM NaNO_2_, Af293) did not reveal substantial differences between the transcriptional activities of these genes (Additional file 2).

Regarding iron metabolism, both *hapX* and *sreA* were upregulated; iron acquisition genes, Siderophore cluster genes, and SidC cluster genes as well were enriched in the upregulated gene set (Additional file 2). Heme binding protein genes (44 out of the studied 121 genes) were also enriched in the upregulated gene set, while Fe-S cluster protein genes (14 out of the studied 43 genes) were enriched in the downregulated gene set (Additional file 2). These changes suggest that the treatment had stronger transcriptional consequences on iron metabolism in the *ΔmeaB* gene deletion mutant than in the reference strain (Additional file 2). The direct comparison of transcriptomes of the treated cultures (*ΔmeaB* vs. Af293) also supported this view: Iron acquisition genes, Siderophore cluster genes, and SidC cluster genes were enriched in the upregulated gene set, while heme binding protein genes were enriched in both the upregulated and the downregulated gene sets (Additional file 2).

In contrast to the Af293 strain, arginine biosynthesis genes were not enriched in the upregulated gene set, however *amcA* encoding a mitochondrial ornithine carrier protein involved in siderophore biosynthesis [61] had significantly higher transcriptional activity in the *ΔmeaB* mutant than in the wild type strain under untreated conditions, and NaNO_2_ treatment further increased this difference (Fig. 5A, Additional file 2). In contrast to the loss of function *meaB6* mutant of *A. nidulans* showing impaired arginine utilization [33], the *ΔmeaB* mutant of *A. fumigatus* could grow on arginine as sole C and N source as fast as the wild type and the complemented strain (Fig. 5D). However, as the photos taken from the colonies placed on a transilluminator implies, the density of the conidiophores was smaller in the case of the gene deletion mutant (Fig. 5D). More importantly, *ΔmeaB* mutant was hypersensitive to NaNO_2_ on arginine even at pH 6.5 (Fig. 5D).

Importantly, deletion of the *meaB* gene did not upregulate the *gnoA* and *fhpA* genes under untreated conditions (Fig. 5A) (i.e., did not cause nitrosative stress). Accordingly, the DCF-test showed only little, while the DAF-FM test (more specific to NO [21]) showed no significant difference between the two strains (Fig. 3B and C). Yet, iron acquisition and Siderophore cluster genes were enriched in the upregulated gene set while heme binding genes were enriched in the downregulated gene set when the transcriptome of the *ΔmeaB* mutant was compared with the Af293 strain under untreated conditions (Additional files 2 and 7).

## Discussion

### Treatment with nitrite induced nitrosative stress in *A. fumigatus*

Our data show that NaNO_2_ treatment caused nitrosative stress in these experiments: The growth inhibitory effect of NaNO_2_ was stronger on acidic pH (Fig. 2) when the formation of RNI from nitrite is stronger [10]. Both the DCF-test and the DAF-FM test indicated disturbed redox homeostasis in NaNO_2_ treated cultures (Fig. 3B and C) without the bulk upregulation of antioxidative enzyme genes (Additional file 2). Note, the DCF-test is not specific for certain reactive oxygen/nitrogen species but detects various oxidizing agents including NO_2_ [64], while DAF-FM test is known to be selective to NO [21]. Upregulation of nitrosative stress response genes, such as *fhpA*, *fhpB*, *gnoA* was observed (Figs. 3D and 5A, Additional file 2). The involvement of these *A. fumigatus* genes in nitrosative stress response was shown previously by the increased nitrosative stress sensitivity of the appropriate single and double gene deletion mutants [15]. Interestingly, GSH metabolism genes (such as *glr1* glutathione reductase, or *Afu3g13900* γ-glutamyl-cisteine ligase and *Afu5g06610* GSH synthase) and thioredoxin metabolism genes (such as *trxA* thioredoxin, and *trxR* thioredoxin reductase) were not among the genes upregulated by the NaNO_2_ treatment in our experiments (Additional file 2), however these genes belong to the GnoA-dependent detoxification of RNI [4, 5]. In *C. neoformans* the *trr1* tioredoxin reductase and the *glr1* glutathione reductase genes [25, 65, 66], and in *C. albicans* the *GLR1* glutathione reductase and *GSH1* glutathione synthase genes [26] were all upregulated under nitrosative stress together with the flavohemoglobin NO dioxygenase gene (*fhb1* and *YHB1*, respectively). In the case of *A. fumigatus*, the very high *fhpA* transcriptional activity suggests that the Fhp pathway was more important than the Gno pathway (Figs. 3D and 5A, Additional file 2), and therefore the high GSH content of *A. fumigatus* cultures [52] may have been adequate for the efficient function of the Gno pathway. This is in line with the observation of Lapp et al. [15], who found that deletion of *fhpA* more strongly increased the nitrosative stress sensitivity of *A. fumigatus* than deletion of *gnoA*. The importance of FhpA in the nitrite dependent nitrosative stress response is also demonstrated by the co-regulation of *fhpA* with the *niaD*, *niiA* (nitrate assimilation) genes in *A. nidulans* [14]. Similar co-regulation may also exist in *A. fumigatus*. The transcriptional activity of *fhpA*, *niaD*, *niiA*, as well as *gnoA* and *fhpB* genes, were 54 ± 31, 15 ± 1, 3 ± 2, 260 ± 8, and 53 ± 2 RPKM, respectively, in a NaNO_3_ containing medium in the absence of any carbon source, though when this medium was supplemented with glucose, the transcriptional activity values increased to 2789 ± 646, 772 ± 77, and 1598 ± 322 RPKM in the case of *fhpA*, *niaD*, and *niiA*, respectively, except in the case of *gnoA* (169 ± 16) and *fhpB* (20 ± 1) where they remained low (Gene Expression Omnibus database; http://www.ncbi.nlm.nih.gov/geo/; accession number: GSE216000 [52]). Co-regulation of nitrate assimilation with flavohemoglobin NO dioxygenase activity can prevent accumulation of RNI during utilization of nitrate as a nitrogen source, and can help to transform nitrite and/or RNI into less dangerous ammonia under nitrosative stress. In line with this, precultivation on nitrate (instead of glutamate) reduced the nitrite sensitivity of *A. fumigatus* (Fig. 5B).

GTP Cyclohydrolase II and HemC were found to be important in nitrosative stress protection in *S. cerevisiae* [41] and *A. nidulans* [67], respectively. In *A. fumigatus*, nitrite did not upregulate the *Afu2g01220* gene encoding a putative GTP Cyclohydrolase II, nor the genes *Afu3g13120* and *Afu5g11760*, orthologues of *A. nidulans* AN0121 (*hemC*; encoding heme-biosynthetic porphobilinogen deaminase) (Additional file 2), suggesting that these genes were not essential under the studied nitrosative stress in this fungus.

In *C. albicans*, other than the *GLR1* and *GSH1* GSH metabolism genes, several GST genes and the *YCF1* glutathione S-conjugate transporter gene were also upregulated under nitrosative stress [26]. In *A. fumigatus*, 7 GST genes were upregulated in the presence of 135 mM NaNO_2_ and these upregulated GST genes showed enrichment when the cultures treated with 135 mM NaNO_2_ and with 90 mM NaNO_2_ were compared (Additional file 2). Understanding the role of GSTs in the nitrosative stress response needs further investigation. Nevertheless, unsaturated fatty acids and even sterols can be oxidized to reactive epoxides that are the substrate of GSTs under stress [68]. Upregulation of GST genes can be the consequence of such oxidation, which is also in line with the upregulation of ergosterol biosynthesis genes (Additional file 2) and the elevated sterol content of NaNO_2_ treated cells (Fig. 5C).

NO, even at low concentrations, reversibly inhibits the activity of cytochrome c oxidase and enhances superoxide production of cytochrome bc1 complex (complex III) [69]. At higher concentrations, NO and other RNI can also irreversibly inactivate all four main complexes of aerobic respiration [69]. Not surprisingly, nitrosative stress affects the transcription of respiration genes. Nitrosative stress upregulated 28 respiration genes in *C. neoformans* [66] and 6 respiration genes including the *AOX1* alternative oxidase gene in *Histoplasma capsulatum* [70]. In *C. albicans*, both of the two alternative oxidase genes (*AOX1* and *AOX2*) were upregulated, while the other 21 respiration genes (cytochrome c, and subunits of complexes I-IV) were downregulated by nitrosative stress [26]. In our experiments, *aox1* showed upregulation under nitrosative stress and five further genes (*cycA* cytochrome c gene and four cytochrome c oxidase genes) were upregulated by the 135 mM NaNO_2_ treatment (Additional file 2). These data suggest that alternative oxidase can be crucial under nitrosative stress: The high alternative oxidase activity can maintain respiration at low cytochrome c oxidase activity and reduces the activity and hence superoxide production of cytochrome bc1 complex.

**Fig. 6:**
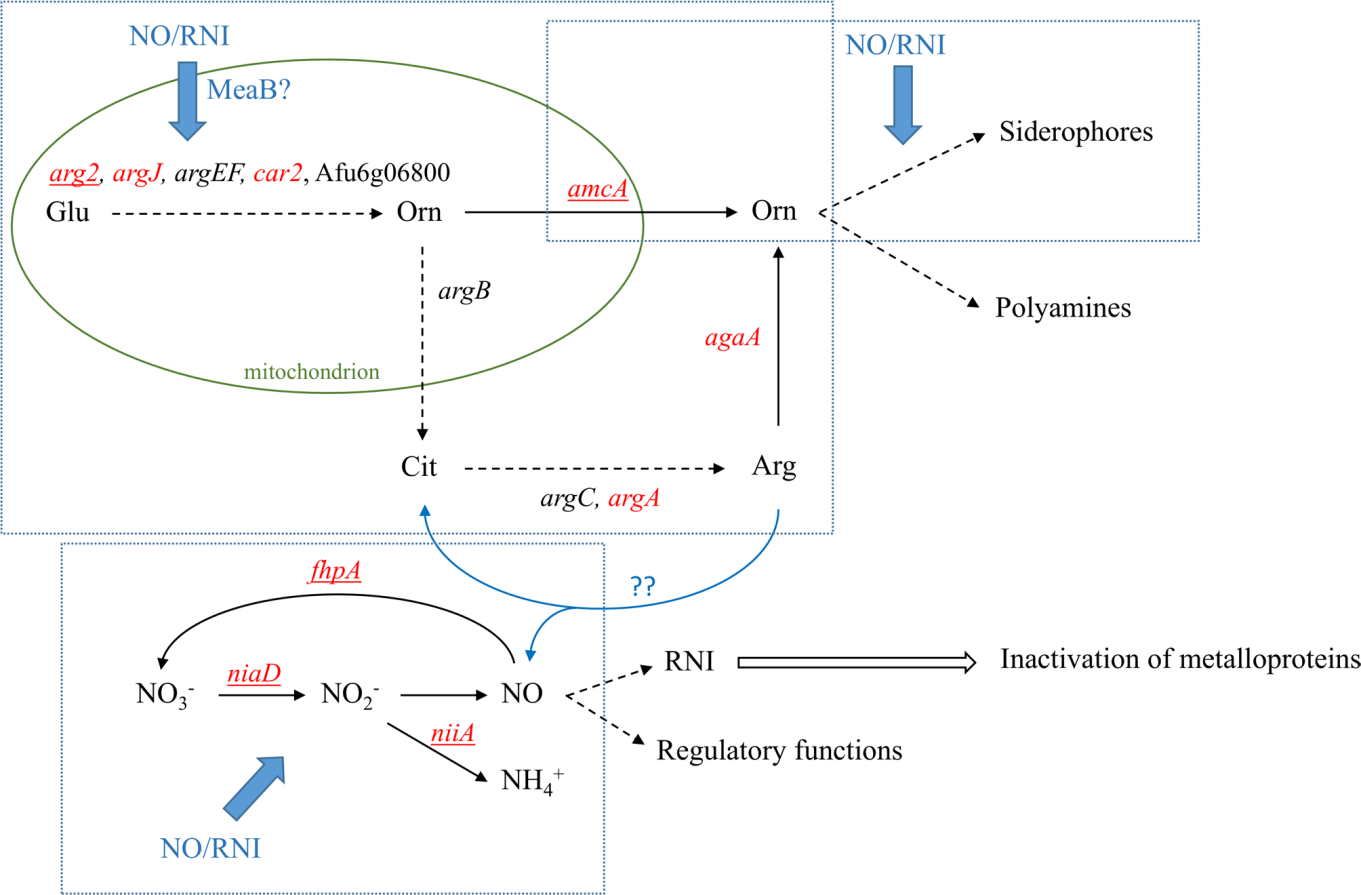
Schematic representation of a possible link between arginine metabolism, NO production and siderophore biosynthesis. Continuous lines represent one step processes; dashed lines indicate the (consecutive) actions of more than one proteins. Glu – glutamate; Orn – ornithine; Cit – citrulline; Arg – arginine *arg2* – acetylglutamate synthase; *argJ* – glutamate N-acetyltransferase; *argEF* – acetylglutamate kinase; *car2* – acetylornithine aminotransferase; *Afu6g06800* – acetylornithine deacetylase; *argB* – ornithine carbamoyltransferase; *argC* – argininosuccinate synthase; *argA* – argininosuccinate lyase; *agaA* – arginase; *amcA* – mitochondrial ornithine carrier protein; *niaD* – nitrate reductase; *niiA* – nitrite reductase; *fhpA* – flavohemoglobin NO dioxygenase. Genes upregulated by nitrosative stress in the *A. fumigatus* Af293 strain or in the VKmeaB1 (*ΔmeaB*) mutant are indicated in red letters or underlined, respectively

Upregulation of iron acquisition genes also appears to be a common response to nitrosative stress: Nitrosative stress upregulated iron uptake genes in *C. neoformans* [66], *H. capsulatum* [70], *C. albicans* [26], and in our observations of *A. fumigatus* (Additional file 2) as well. In *A. fumigatus*, upregulation of RIA genes (*ftrA* and *fetC*), siderophore metabolism (both synthesis and ferri-siderophore transport) genes as well as *hapX* encoding a transcription factor upregulating iron uptake genes [51] were seen (Additional file 2). These upregulations were accompanied by the enrichment of heme binding protein genes in the upregulated gene set (Additional file 2). NO and other RNI can inactivate metalloproteins [12]. Re-synthesis of these proteins can be supported by enhanced iron uptake and upregulation of metalloprotein genes which may explain the observed transcriptional changes. In addition, intracellular siderophores can safely store iron [51], therefore upregulation of siderophore biosynthesis genes can also prepare cells to protect themselves against the free iron liberated from the damaged metalloproteins. Upregulation of *amcA* encoding a mitochondrial ornithine transporter is particularly interesting (Additional file 2), since it connects iron metabolism (siderophore formation) to arginine biosynthesis (Fig. 6). Schafferer et al. [61] found that siderophore biosynthesis depends on mitochondrial ornithine formation and the AmcA dependent transport of ornithine to the cytosol rather than production of ornithine *via* arginine (Fig. 6), therefore arginine and siderophore synthesis are competing with each other. According to our results (Additional file 2, Fig. 6), nitrite elicited nitrosative stress activates genes of arginine metabolism that may shift ornithine from arginine biosynthesis to siderophore production. This could contribute the maintenance of iron homeostasis under nitrosative stress (Fig. 6).

### MeaB may affect stress tolerance via arginine metabolism

Deletion of the *meaB* bZIP transcription factor gene increased nitrite sensitivity in *A. fumigatus* Af293 (Fig. 2), demonstrating that MeaB is involved in nitrosative stress response. The substantial differences between the transcriptome detected in the presence of 90 mM NaNO_2_ in the two strains and the transcriptional changes caused by the treatment with 90 mM NaNO_2_ in the wild type and the *ΔmeaB* strains (Fig. 4, Additional files 1) would suggest that MeaB takes part in the regulation of many elements of the nitrosative stress response. However, the differences between the behaviors of the two strains decreased markedly when the cultures of the wild type strain treated with elevated (135 mM) concentration of nitrite was compared with cultures of the gene deletion mutant treated with 90 mM NaNO_2_ (Additional file 1, Figs. 3, 4 and 5C). This pattern implies that the *ΔmeaB* strain responded to the nitrite stress very similarly to the wild type strain, but for the mutant the stress was stronger when the stressor was added to the two types of cultures in the same concentration. The changes detected in the DCM and the observed redox imbalance (DCF-test) after the treatments also support the idea that 90 mM NaNO_2_ induced larger stress in the *ΔmeaB* mutant than in the reference strain (Fig. 3).

To explain the phenotype of the *ΔmeaB* strain, we assume that MeaB as a transcription factor of nitrogen metabolism [33, 34] is important in the fine tuning of the transcription of arginine metabolism genes. This is supported by the altered transcriptional levels of arginine metabolism genes detected in the two strains (Fig. 5A, Additional file 2). Arginine metabolism can be important for fungi for different reasons. (i) NO can be formed from arginine by NOS [6]. Although this process is well known in mammals, fungal arginine dependent NO production is less known [5, 7–9], however a NOS-like activity and arginine dependent NO formation was demonstrated in *A. nidulans* [5, 7]. (ii) Arginine metabolism also affects polyamine formation which is started with decarboxylation of ornithine [71]. Polyamines are known to be important in maintaining redox homeostasis in fungi, however this field also appears to be understudied [72]. (iii) Siderophore production of *A. fumigatus* also needs ornithine, and a proper iron metabolism is crucial for appropriate redox balance [61, 71].

Although there is no direct evidence of arginine-dependent (NOS-dependent) NO formation in *A. fumigatus*, Oiki et al. [21] found that *A. fumigatus* produces NO under various stresses irrespective of the available nitrogen sources (nitrate, ammonium or proline) suggesting that other sources of NO than nitrite should occur. Some of our results also raise the possibility of the occurrence of nitrite independent NO production in this fungus: The lack of MeaB increased MSB (but not tBOOH or H_2_O_2_) stress sensitivity of the strain (Fig. 2, Additional file 4). This phenotype may also be linked to nitrosative stress, since superoxide generated under MSB stress [73] can form toxic peroxynitrite with NO [12, 13]. An altered endogen NO production therefore may lead to increased sensitivity against superoxide generating agents. The reduced conidiophore formation on arginine as the sole carbon and nitrogen source observed with the ***Δ****meaB* gene deletion mutant (Fig. 5D) also supports the view that MeaB may affect endogen (arginine dependent/nitrite independent) NO production, since elevated NO levels inhibit asexual development in *Aspergillus* species [17, 19]. Moreover, the nitrite sensitivity was stronger on arginine as sole C and N source than on glucose (Figs. 2A and 5D).

On the other hand, our transcriptomic data show that transcription of ornithine decarboxylase gene (*odcA*; [71]) was not affected by deletion of the *meaB* gene and strong nitrosative stress even downregulated it in both the wild type and the *ΔmeaB* strains (Fig. 5A; Additional file 2). Nevertheless, alterations in arginine metabolism caused by the lack of MeaB may affect stress tolerance attributes of *A. fumigatus via* polyamines.

The Siderophore cluster genes were upregulated both in the lack of MeaB and by nitrite treatment (Fig. 5A; Additional file 2) suggesting alterations in iron homeostasis. This can affect nitrosative and oxidative stress sensitivity of metalloproteins and hence stress tolerance of the fungus.

The *ΔmeaB* strain (originated from *ΔakuB*^*KU80*^) used in a previous study [32] was characterized with reduced growth, biofilm formation, and galactosaminogalactan content, attenuated in vivo virulence (in *G. mellonella* infection model), as well as increased CR, calcofluor white, sodium dodecyl sulfate, hyperosmotic and heat stress sensitivity. The lack of MeaB downregulated Amino sugar and nucleotide sugar metabolism, Tyrosine metabolism, Iron ion homeostasis genes, and several other genes involved in cell wall biogenesis. In contrast, our *ΔmeaB* strain (originated from the Af293) did not show reduced growth (Fig. 1) or altered in vivo virulence (Additional file 5) and its hyperosmotic or CR stress tolerance decreased only moderately (Fig. 2). The lack of MeaB downregulated amino sugar catabolic process, and chitinase genes, while upregulated iron acquisition, and glucanase genes (Additional files 2 and 6). These differences may suggest that the MeaB influenced the above mentioned processes mainly indirectly. Considering that NO is an important signal transduction molecule [4, 5, 17–21], the nitrosative stress tolerance of the two parental strains (*ΔakuB*^*KU80*^ and Af293) was different (Additional file 3), and the absence of MeaB reduced nitrosative stress tolerance (in strain Af293; Fig. 2), one of the possible explanations for the differences observed between the two mutants is that their NO homeostasis was different.

## Conclusions

In short, we assume that MeaB is important in the fine-tuning the transcriptional activity of arginine metabolism genes. Lack of MeaB disturbs arginine metabolism, which has multiple consequences on the physiology of the fungus since arginine metabolism contributes to siderophore production (iron homeostasis), polyamine formation (stress tolerance) and may also affect NO homeostasis. Our results also suggest that, although inhibition of nitrosative stress defense in *A. fumigatus* may not be an effective antifungal therapy for all *A. fumigatus* strains, a combined approach based on disruption of iron and NO homeostasis is promising. Generation of nitrosative (nitrooxidative) stress in the fungus increases the demand for iron, which can enhance the effectiveness of antifungal strategies aimed at inhibiting iron acquisition by the fungus.

## Supplementary Information

Supplementary Material 1.

Supplementary Material 2.

Supplementary Material 3.

Supplementary Material 4.

Supplementary Material 5.

Supplementary Material 6.

Supplementary Material 7.

## Data Availability

The transcriptome data sets are available in the Gene Expression Omnibus database (GEO; http://www.ncbi.nlm.nih.gov/geo/) with the following accession number: GSE277801.

## Abbreviations

CAZyme: carbohydrate-active enzyme
CP: crossing point
CR: Congo Red
DCF: 2’,7’-dichlorofluorescein
DCM: dry cell mass
DEG: differentially expressed genes
DFP: deferiprone
fhb: flavohemoglobin NO dioxygenase
GM-CSF: granulocyte-macrophage colony-stimulating factor
Gno: S-nitrosoglutathione reductase
GSH: (reduced) glutathione
GSNO: S-nitrosoglutathione
M-CSF: macrophage colony-stimulating factor
MSB: menadione sodium bisulfite
NOS: NO synthase
RIA: reductive iron assimilation
RNI: reactive nitrogen intermediates
ROS: reactive oxygen species
RT-qPCR: reverse-Transcriptional Quantitative Real-Time Polymerase Chain Reaction
tBOOH: *terc*-butyl-hydroperoxide
TCA cycle: tricarboxylic acid cycle
