# Supplementary material for: The MeaB bZIP transcription factor is needed for proper nitrosative stress response induced by nitrite in Aspergillus fumigatus
Source: BMC Genomics. 2025 Sep 29;26:849. doi: 10.1186/s12864-025-11990-3 (PMC12482460; doi:10.1186/s12864-025-11990-3)
Supplement: Supplementary file 3 — Supplementary Material 3. [file 12864_2025_11990_MOESM3_ESM.pptx]

## Slide 1
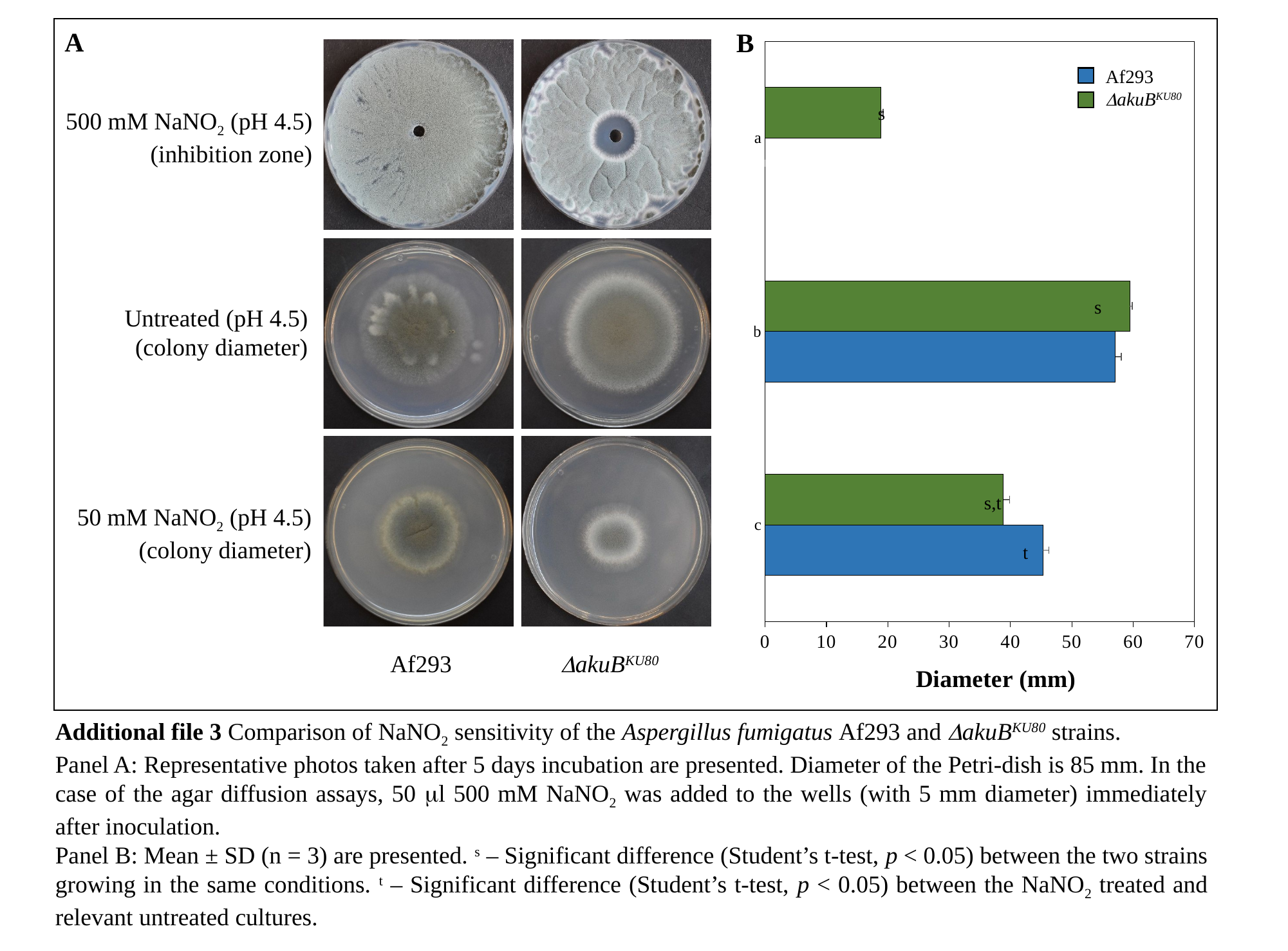

### Chart
| Category | Af293 | AkuB |
|---|---|---|
| c | 45.29040404040404 | 38.851010101010104 |
| b | 57.095959595959606 | 59.4570707070707 |
| a | 0.0 | 18.88888888888889 |A
B
Af293
DakuBKU80
s
500 mM NaNO2 (pH 4.5)
(inhibition zone)
s
Untreated (pH 4.5)
(colony diameter)
s,t
50 mM NaNO2 (pH 4.5)
(colony diameter)
t
Af293
DakuBKU80
Additional file 3 Comparison of NaNO2 sensitivity of the Aspergillus fumigatus Af293 and DakuBKU80 strains.
Panel A: Representative photos taken after 5 days incubation are presented. Diameter of the Petri-dish is 85 mm. In the case of the agar diffusion assays, 50 ml 500 mM NaNO2 was added to the wells (with 5 mm diameter) immediately after inoculation.
Panel B: Mean ± SD (n = 3) are presented. s – Significant difference (Student’s t-test, p < 0.05) between the two strains growing in the same conditions. t – Significant difference (Student’s t-test, p < 0.05) between the NaNO2 treated and relevant untreated cultures.
