# Supplementary material for: The MeaB bZIP transcription factor is needed for proper nitrosative stress response induced by nitrite in Aspergillus fumigatus
Source: BMC Genomics. 2025 Sep 29;26:849. doi: 10.1186/s12864-025-11990-3 (PMC12482460; doi:10.1186/s12864-025-11990-3)
Supplement: Supplementary file 4 — Supplementary Material 4. [file 12864_2025_11990_MOESM4_ESM.pptx]

## Slide 1
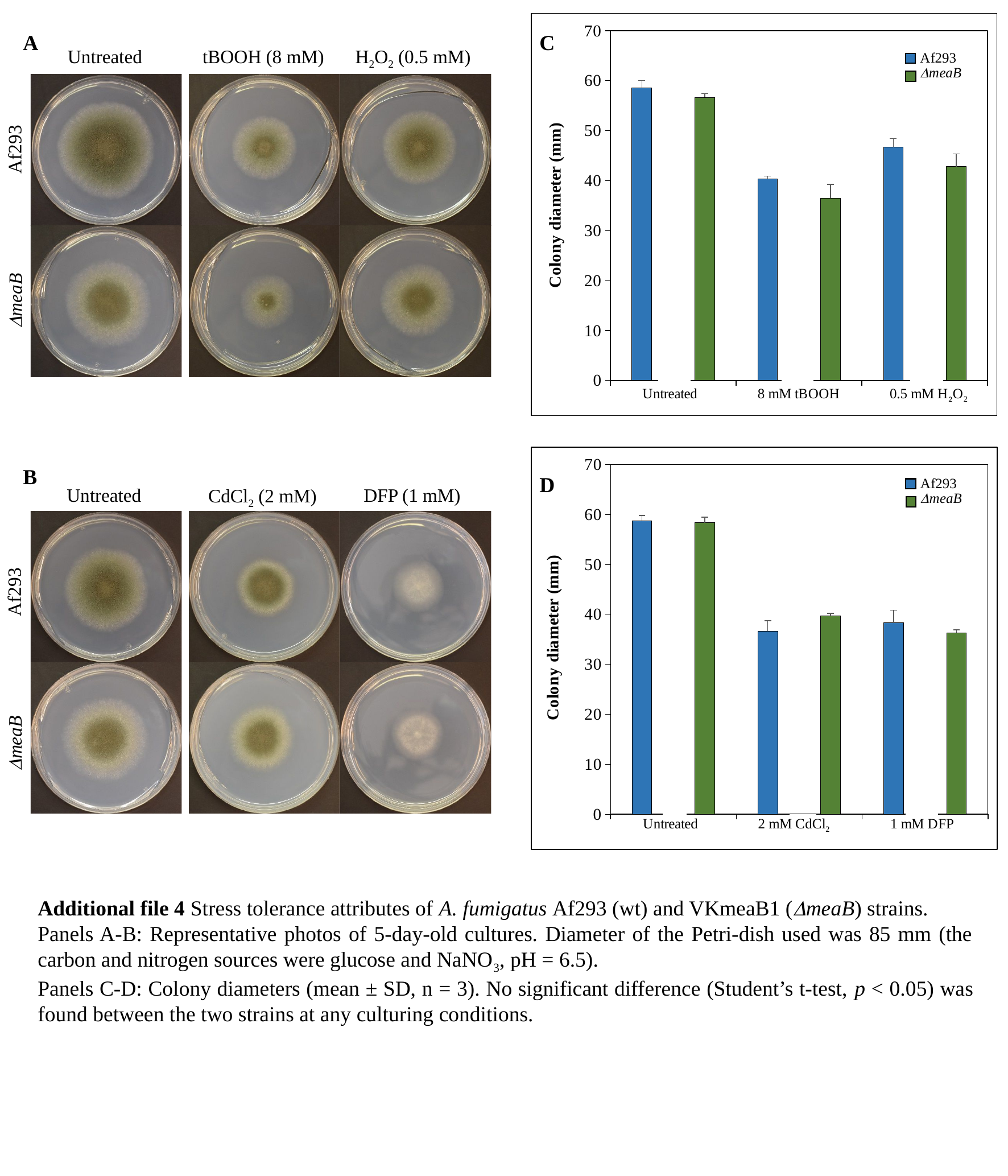

### Chart
| Category | x |
|---|---|
| untr | 58.5 |
| untr | 56.666666666666664 |
| tBOOH | 40.333333333333336 |
| tBOOH | 36.5 |
| H2O2 | 46.666666666666664 |
| H2O2 | 42.833333333333336 |A
C
H2O2 (0.5 mM)
Untreated
tBOOH (8 mM)
Af293
DmeaB
### Chart
| Category | x |
|---|---|
| untr | 58.666666666666664 |
| untr | 58.333333333333336 |
| CdCl2 | 36.666666666666664 |
| CdCl2 | 39.666666666666664 |
| DFP | 38.333333333333336 |
| DFP | 36.333333333333336 |B
D
DFP (1 mM)
Untreated
CdCl2 (2 mM)
Af293
DmeaB
Additional file 4 Stress tolerance attributes of A. fumigatus Af293 (wt) and VKmeaB1 (DmeaB) strains.
Panels A-B: Representative photos of 5-day-old cultures. Diameter of the Petri-dish used was 85 mm (the carbon and nitrogen sources were glucose and NaNO3, pH = 6.5).
Panels C-D: Colony diameters (mean ± SD, n = 3). No significant difference (Student’s t-test, p < 0.05) was found between the two strains at any culturing conditions.
