# Supplementary material for: The MeaB bZIP transcription factor is needed for proper nitrosative stress response induced by nitrite in Aspergillus fumigatus
Source: BMC Genomics. 2025 Sep 29;26:849. doi: 10.1186/s12864-025-11990-3 (PMC12482460; doi:10.1186/s12864-025-11990-3)
Supplement: Supplementary file 5 — Supplementary Material 5. [file 12864_2025_11990_MOESM5_ESM.pptx]

## Slide 1
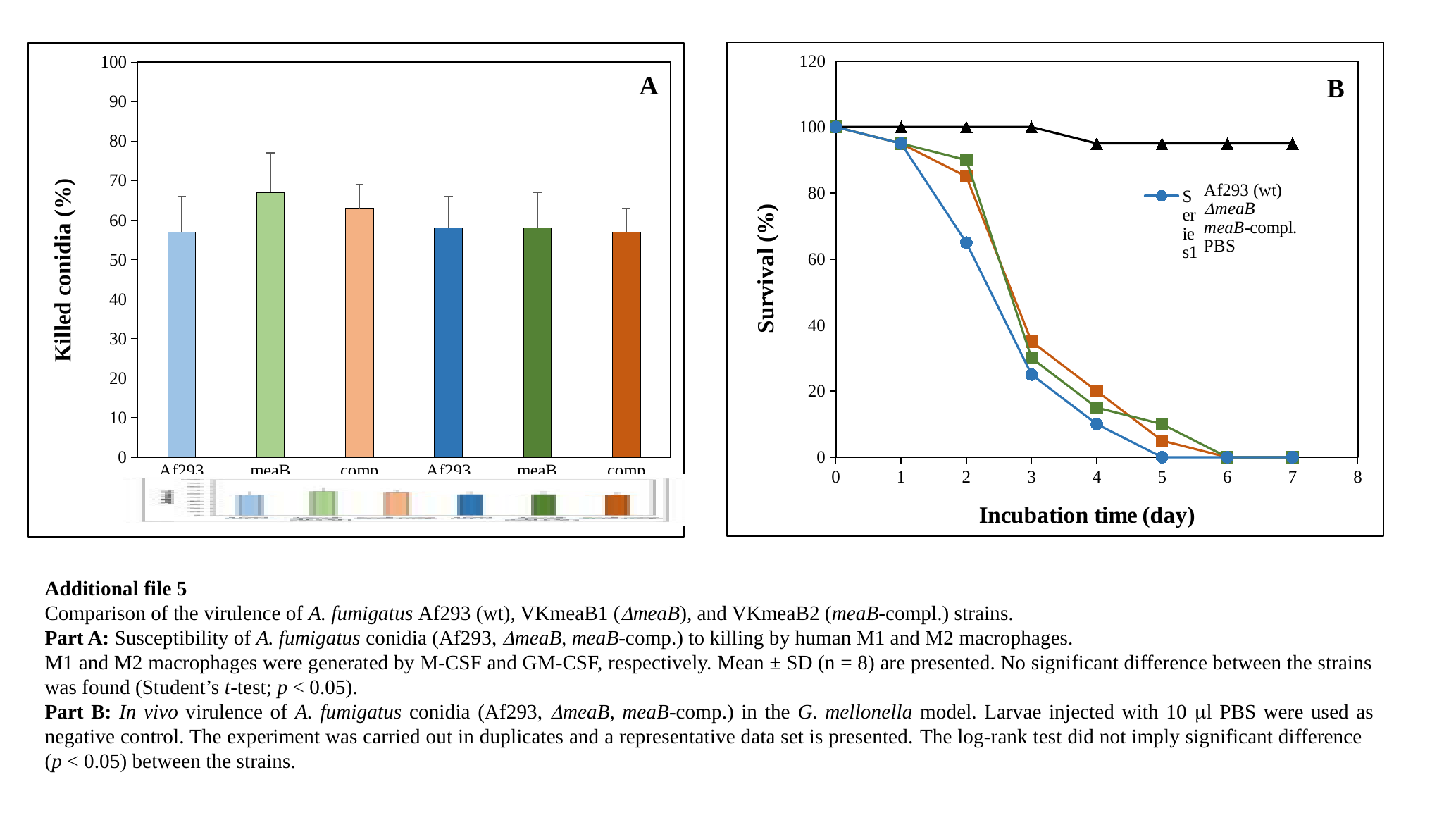

### Chart
| Category | | | | |
|---|---|---|---|---|
### Chart
| Category | kill |
|---|---|
| Af293 | 57.0 |
| meaB | 67.0 |
| comp | 63.0 |
| Af293 | 58.0 |
| meaB | 58.0 |
| comp | 57.0 |A
B
Additional file 5
Comparison of the virulence of A. fumigatus Af293 (wt), VKmeaB1 (DmeaB), and VKmeaB2 (meaB-compl.) strains.
Part A: Susceptibility of A. fumigatus conidia (Af293, DmeaB, meaB-comp.) to killing by human M1 and M2 macrophages.
M1 and M2 macrophages were generated by M-CSF and GM-CSF, respectively. Mean ± SD (n = 8) are presented. No significant difference between the strains was found (Student’s t-test; p < 0.05).
Part B: In vivo virulence of A. fumigatus conidia (Af293, DmeaB, meaB-comp.) in the G. mellonella model. Larvae injected with 10 ml PBS were used as negative control. The experiment was carried out in duplicates and a representative data set is presented. The log-rank test did not imply significant difference (p < 0.05) between the strains.
