# Supplementary material for: The MeaB bZIP transcription factor is needed for proper nitrosative stress response induced by nitrite in Aspergillus fumigatus
Source: BMC Genomics. 2025 Sep 29;26:849. doi: 10.1186/s12864-025-11990-3 (PMC12482460; doi:10.1186/s12864-025-11990-3)
Supplement: Supplementary file 7 — Supplementary Material 7. [file 12864_2025_11990_MOESM7_ESM.pptx]

## Slide 1
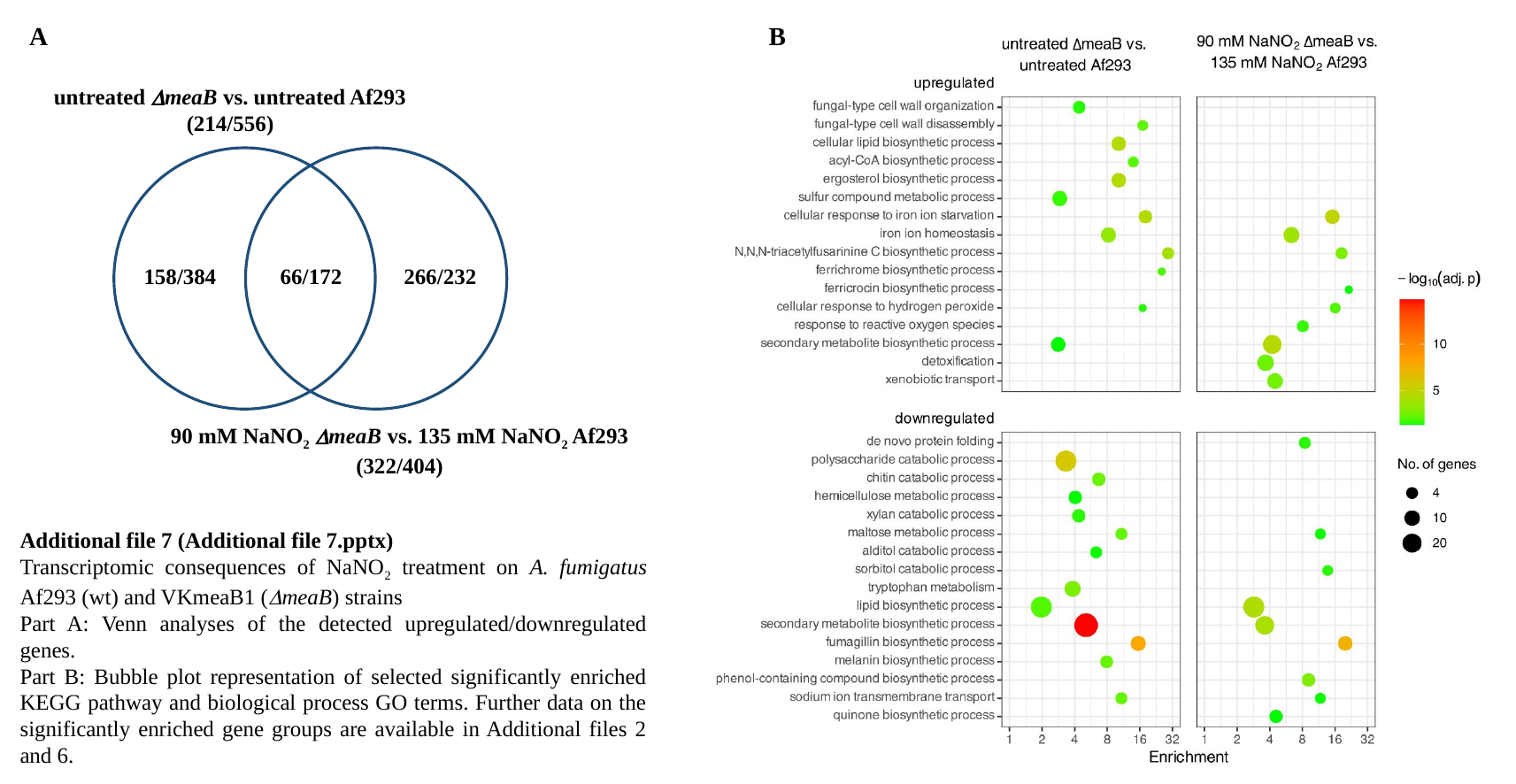

B
A
untreated DmeaB vs. untreated Af293
(214/556)
266/232
158/384
66/172
90 mM NaNO2 DmeaB vs. 135 mM NaNO2 Af293
(322/404)
Additional file 7 (Additional file 7.pptx)
Transcriptomic consequences of NaNO2 treatment on A. fumigatus Af293 (wt) and VKmeaB1 (DmeaB) strains
Part A: Venn analyses of the detected upregulated/downregulated genes.
Part B: Bubble plot representation of selected significantly enriched KEGG pathway and biological process GO terms. Further data on the significantly enriched gene groups are available in Additional files 2 and 6.
